# A rare case of infected urachal cyst leading to intestinal obstruction in a 3-month-old boy with febrile urinary tract infection: Case report

**DOI:** 10.1097/MD.0000000000038615

**Published:** 2024-06-14

**Authors:** Ji Yeon Song, Soo-Hong Kim

**Affiliations:** a Department of Pediatrics, Pusan National University Children’s Hospital, Yangsan, Korea; b School of Medicine, Pusan National University, Busan, Korea; c Division of Pediatric Surgery, Department of Surgery, Pusan National University Yangsan Hospital and Pusan National University Children’s Hospital, Yangsan, Korea.

**Keywords:** abdominal distension, case report, intestinal obstruction, pediatrics, urachal cyst, urinary tract infection

## Abstract

**Rationale:**

Urachal anomalies are rare and can present with various clinical manifestations. Urachal remnants, in particular, can be difficult to diagnose because of atypical symptoms at presentation. This study reports a case of intestinal obstruction in an infant secondary to an infected urachal cyst.

**Patients concerns:**

A 3-month-old boy with a known febrile urinary tract infection developed acute abdominal distension

**Diagnoses:**

Abdominal ultrasound (US) and computed tomography (CT) revealed a nonspecific, ill-defined soft tissue density at the mid-abdomen, associated with intestinal obstruction.

**Interventions:**

Emergency exploratory laparotomy was performed. The site of the obstruction was found to be at the mid-small bowel; the proximal small bowel was markedly distended, and the small bowel and sigmoid colon were adherent to urachal remnant. The urachal remnant was excised, and the peritoneal adhesions were lysed.

**Outcomes:**

The day after surgery, the patient was discharged without any complications.

**Lessons:**

Intestinal obstruction is an exceedingly rare presentation of urachal remnants. This case highlights that urachal anomalies should be considered in the differential diagnosis in patients with intestinal obstruction and a concurrent febrile urinary tract infection.

## 1. Introduction

The urachus serves as a connection between the fetal bladder and allantois.^[1]^ Urachal anomalies are rare diseases that may be congenital or acquired.^[2]^ They are rare and observed in 1 out of 5000 individuals in the general population.^[1]^ While urachal anomalies are mostly asymptomatic, urachal remnants can lead to inflammation, infection, abdominal pain, and even malignancy.^[3]^ This report describes a rare case of a pediatric patient with a urinary tract infection who had intestinal obstruction caused by an infected urachal remnant that presented as abdominal distension. The differential diagnosis for patients presenting with acute abdomen is extensive. When patients with urinary tract infections suddenly develop intestinal obstruction of unknown origin, a urachal remnant should be considered in the differential diagnosis.

## 2. Case presentation

**Figure 1.**
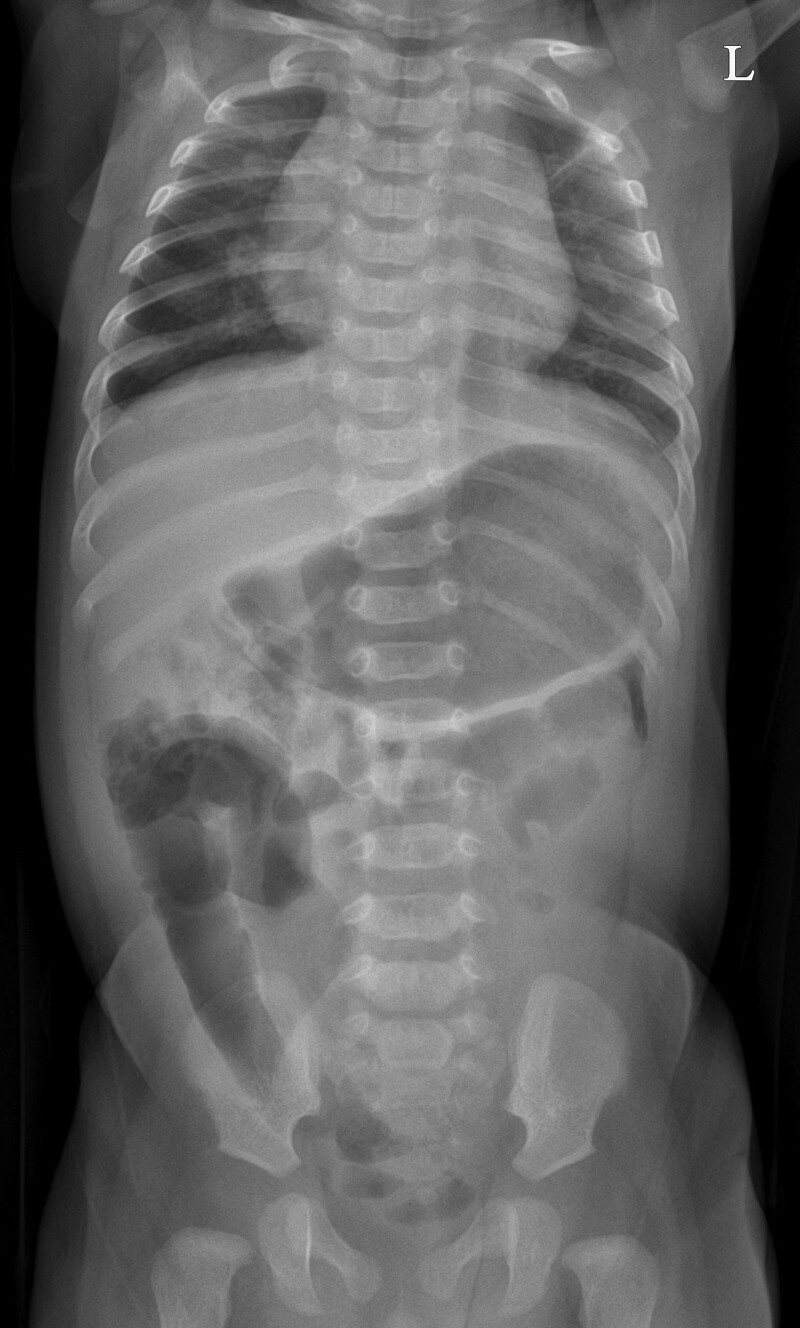
Initial X-ray of the abdomen showed no abnormalities.

**Figure 2.**
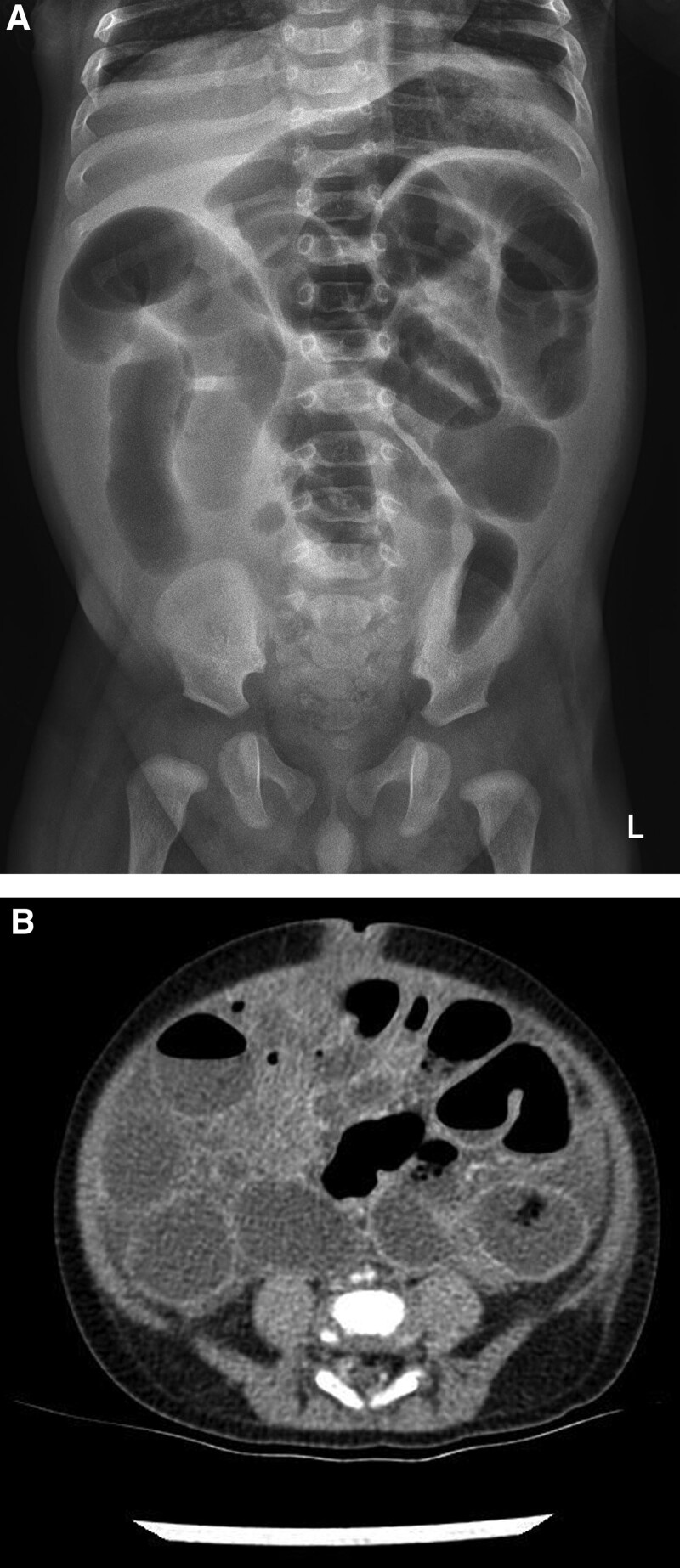
(A) Follow-up X-ray of the abdomen demonstrating small bowel ileus, and (B) computed tomography showing an ill-defined soft tissue density measuring approximately 4.5 × 4.3 cm in the mid-abdomen with associated intestinal obstruction (Day 2).

**Figure 3.**
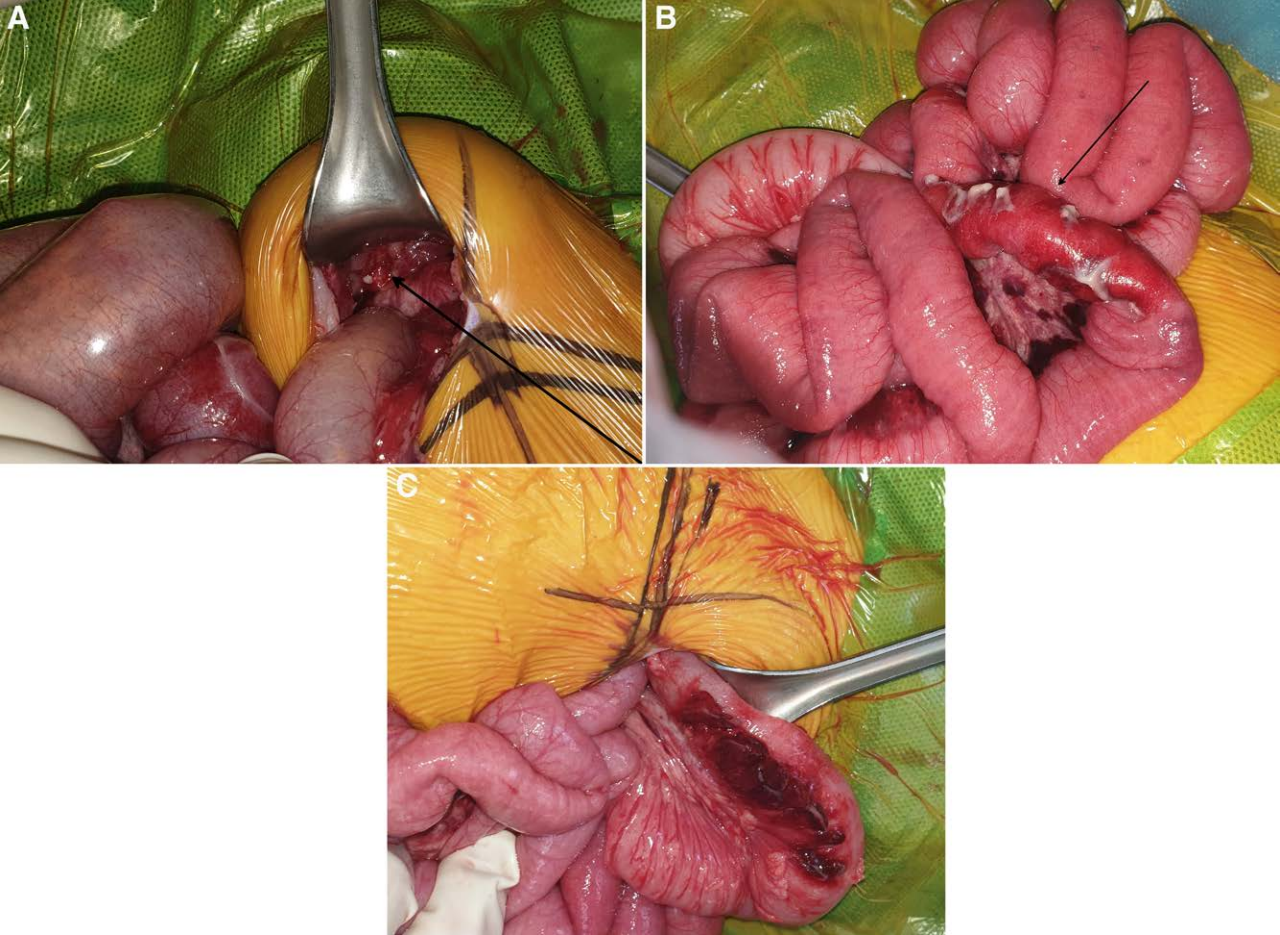
Intraoperative images of (A) urachal remnant abscess (arrow), (B) small bowel (triggering point, arrow), and (C) sigmoid colon.

**Figure 4.**
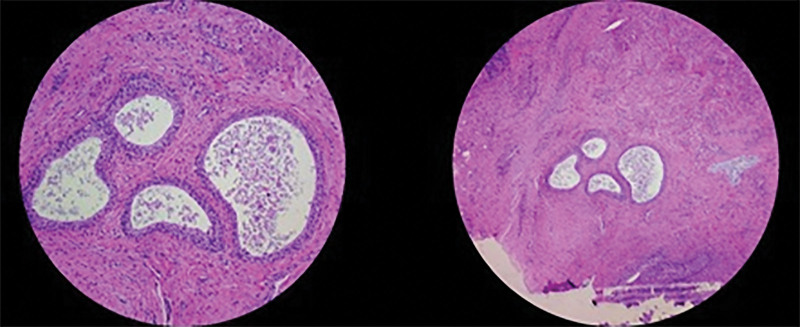
Pathological findings showing urachal cystic remnants lined by benign cuboidal cells and urothelial cells (H&E, ×100).

A 3-month-old boy was presented to the emergency department with poor oral intake and decreased activity. He was born at 40 weeks and 2 days gestation and weighed 3.14 kg. He was previously healthy, and gastrointestinal symptoms, such as projectile vomiting and diarrhea, had not been reported. Physical examination was suggestive of dehydration, and his abdomen was flat and soft. Initial vital signs were as follows: blood pressure, 90/60 mm Hg; heart rate, 180 beats/min; respiratory rate, 30/min; temperature, 38.1°C; and oxygen saturation, 100%. Initial laboratory findings were as follows: white blood cell (WBC) count, 23,040/µL; hemoglobin level, 8.8 g/dL; platelet count, 568,000/µL; C-reactive protein level, 4.75 mg/dL; and procalcitonin level, 1.466 ng/mL. Urinalysis results were as follows: specific gravity, 1.010; WBC count, 10 to 19/high-power field (HPF); red blood cell count, <1/HPF; nitrite concentration, 1+; and leukocyte esterase level, 2+. Urine culture revealed *Klebsiella aerogenes* (>100,000 colonies/mL). The patient was administered intravenous ampicillin-sulbactam for his urinary tract infection. An initial X-ray of the abdomen showed no abnormalities (Fig. 1). On Day 2 of admission, he developed severe abdominal distension with decreased urine output and food intake, had no bowel movement, and appeared drowsy. Follow-up abdominal X-ray demonstrated small bowel ileus that was likely caused by intestinal obstruction (Fig. 2A). Both abdominal ultrasound (US) and computed tomography (CT) revealed a nonspecific, ill-defined soft tissue density at the mid-abdomen that measured approximately 4.5 × 4.3 cm and was associated with intestinal obstruction (Fig. 2B). The triggering point for the intestinal obstruction could not be identified on imaging. Emergency exploratory laparotomy was performed. The site of the obstruction was found to be at the mid-small bowel; the proximal small bowel was markedly distended, and the small bowel and sigmoid colon were adherent to a 2-cm mass, which had triggered adhesional obstruction (Fig. 3). The mass was excised, and the peritoneal adhesions were lysed. No postoperative complications were observed, and a follow-up abdominal X-ray showed improvement in the ileus. The day after surgery, the patient was discharged without any complications. The excised mass was confirmed on pathologic examination to be urachal remnants composed of cysts lined by benign cuboidal cells and urothelial cells (Fig. 4).

## 3. Discussion

Congenital urachal anomalies are typically identified in childhood, particularly in patients where the urachus fails to obliterate.^[4]^ The anomalies consist of the following 4 subtypes: urachal cyst, patent urachus, urachal sinus, and urachal diverticulum.^[1]^ Congenital anomalies are observed in 1.6% of children under 15 years of age and in 0.63% of adults.^[5]^ Acquired anomalies may be associated with inflammation and neoplasm.^[4]^ Most abnormal urachal findings are asymptomatic and detected incidentally during radiographic studies or abdominal surgery.^[6]^ Urachal anomalies have a low incidence (1:5000) in the general population^[1]^; however, researchers have recently reported a higher incidence than that in previous reports (1:1000).^[7]^

Urachal anomalies are more common in men than in women and can have various clinical presentations, such as a palpable umbilical mass, umbilical erythema, abdominal pain, urinary symptoms (e.g., hematuria, dysuria, intermittent urinary retention, and recurrent urinary infections), abdominal distension, and malignancy.^[6]^ Intestinal obstruction secondary to an infected urachal cyst is extremely rare and may represent a chronic process that causes adhesions, such as that reported in this case. There have been a few published reports of intestinal obstruction secondary to infected urachal remnants in adults; however, it is exceedingly rare in childhood. History and physical examination are necessary for the diagnosis of urachal lesions, and a definitive diagnosis requires imaging studies; the specific imaging modalities needed are determined on a case-by-case basis. In general, US and CT are the best diagnostic tools.^[8]^ US is the most common modality for evaluating urachal anomalies in the pediatric population, followed by CT, magnetic resonance imaging, and voiding cystourethrography.^[9]^ US and CT are better suited for detecting urachal anomalies because the urachus is located anterior to the intestinal structures near the abdominal wall.^[10]^ Of note, US has a higher diagnostic accuracy than CT.^[10]^ The diagnosis of urachal anomalies often requires additional radiographic studies to supplement the US findings. However, urachal anomalies cannot be definitively diagnosed using imaging studies but must be confirmed with surgery.

This was a rare case of a patient with a known urinary tract infection confirmed by exploratory laparotomy to have an intestinal obstruction secondary to an infected urachal cyst. Although uncommon, infected urachal remnants should be considered as a potential cause of intestinal obstruction in pediatric patients who present with abdominal distension in the setting of urinary tract infection, especially in patients without a history of abdominal surgery. US is the first imaging modality recommended for the pediatric population. Should the results be inconclusive, CT may be used next.^[10]^

In conclusion, although urachal anomalies are rare, they have the potential to cause an acute abdomen. Clinicians should consider the possibility of infected urachal remnants in patients who present to the emergency department with an acute abdomen.

## Author contributions

**Conceptualization:** Ji Yeon Song, Soo-Hong Kim.

**Data curation:** Ji Yeon Song, Soo-Hong Kim.

**Formal analysis:** Ji Yeon Song, Soo-Hong Kim.

**Funding acquisition:** Ji Yeon Song, Soo-Hong Kim.

**Investigation:** Soo-Hong Kim.

**Methodology:** Soo-Hong Kim.

**Project administration:** Soo-Hong Kim.

**Visualization:** Soo-Hong Kim.

**Writing – original draft:** Ji Yeon Song, Soo-Hong Kim.

**Writing – review & editing:** Ji Yeon Song, Soo-Hong Kim.
